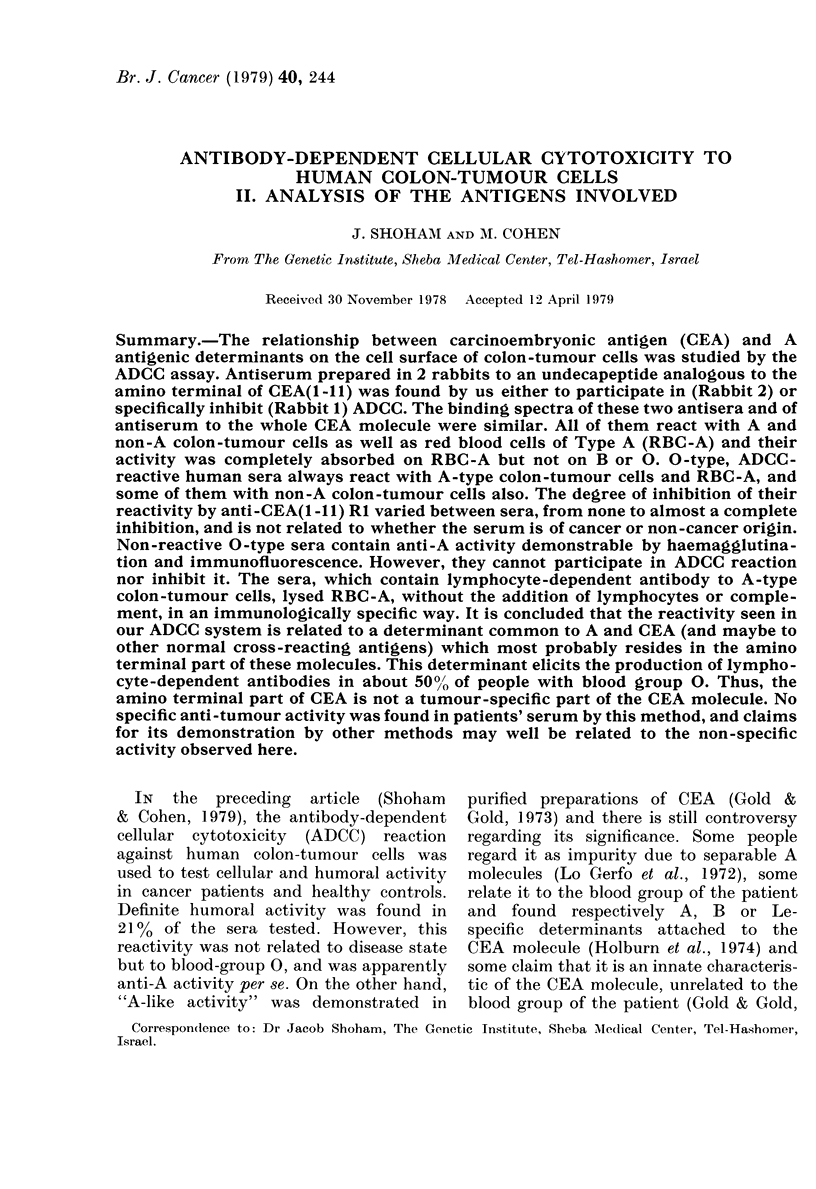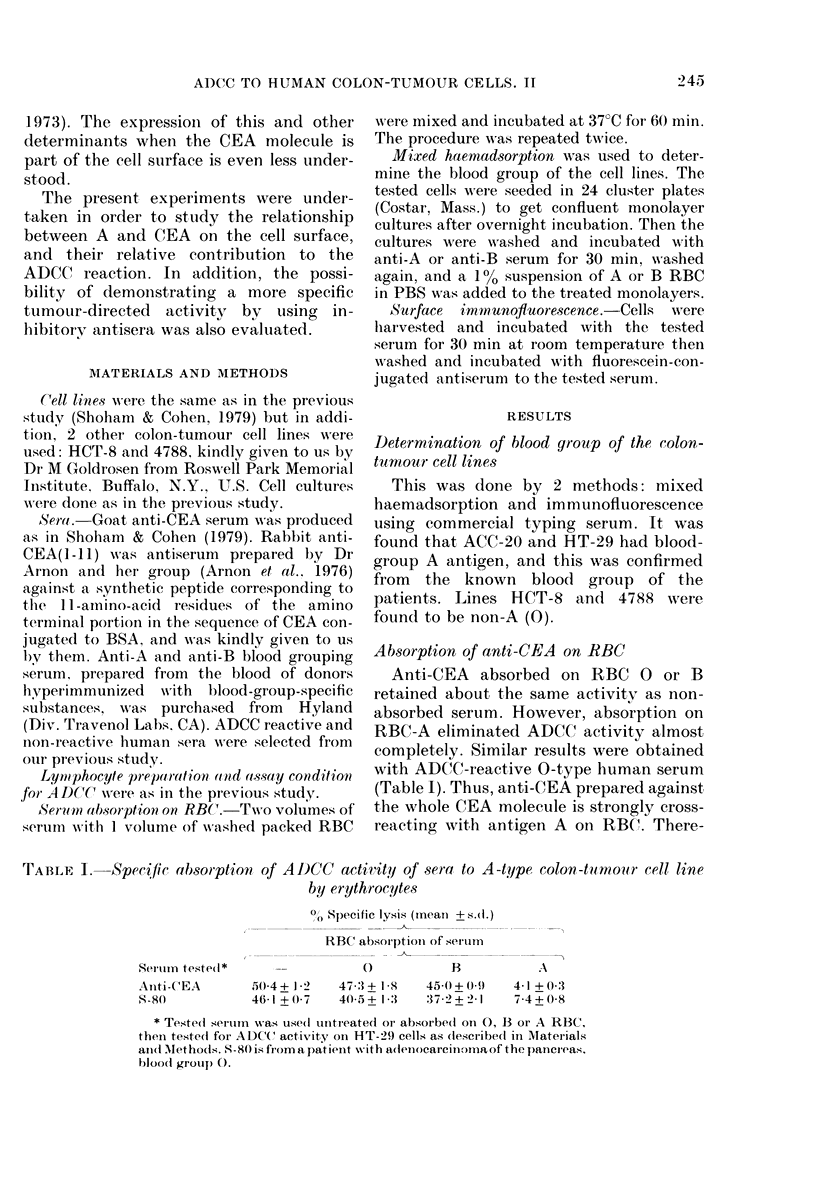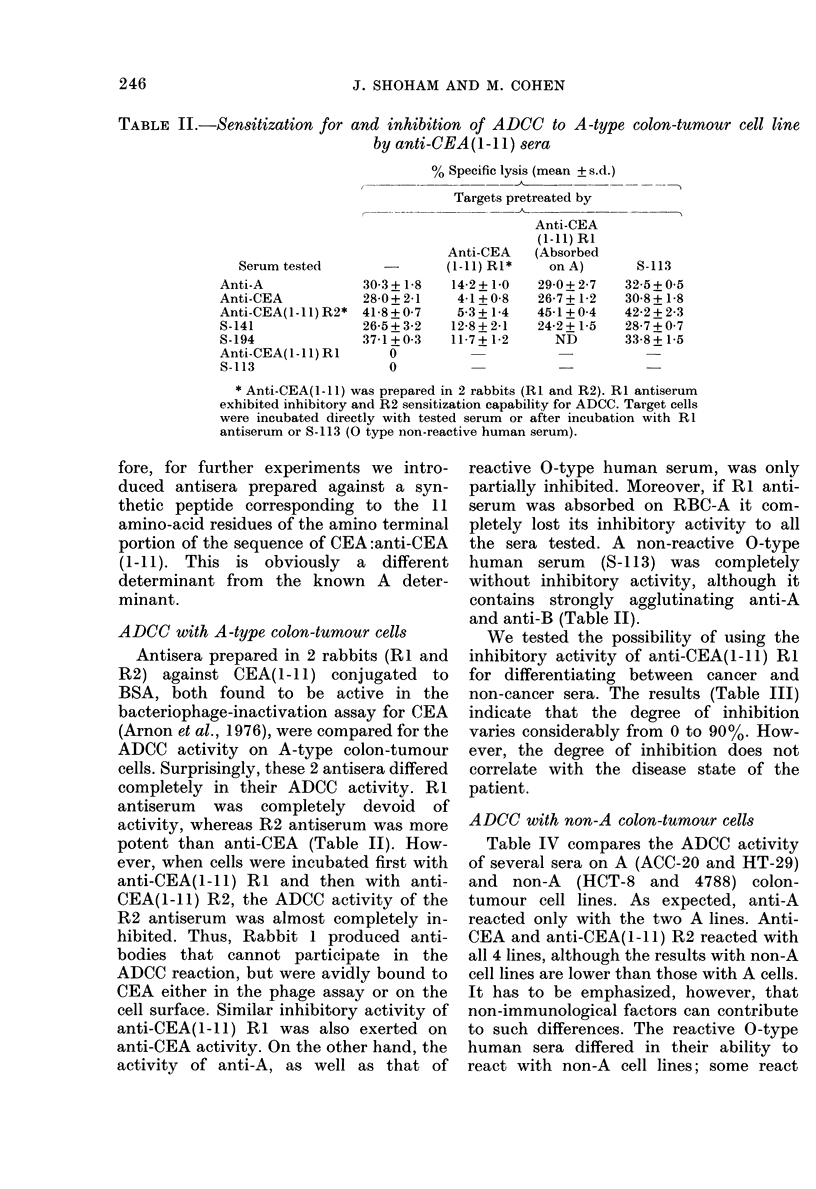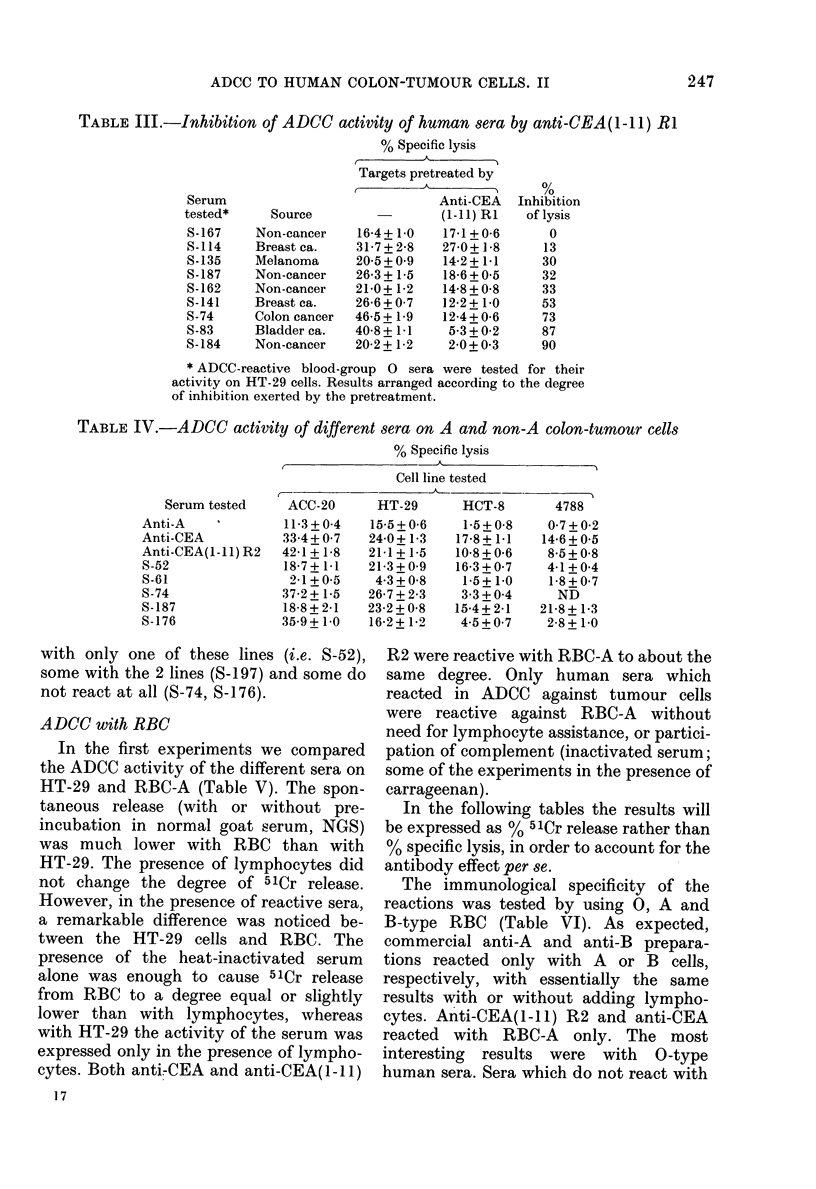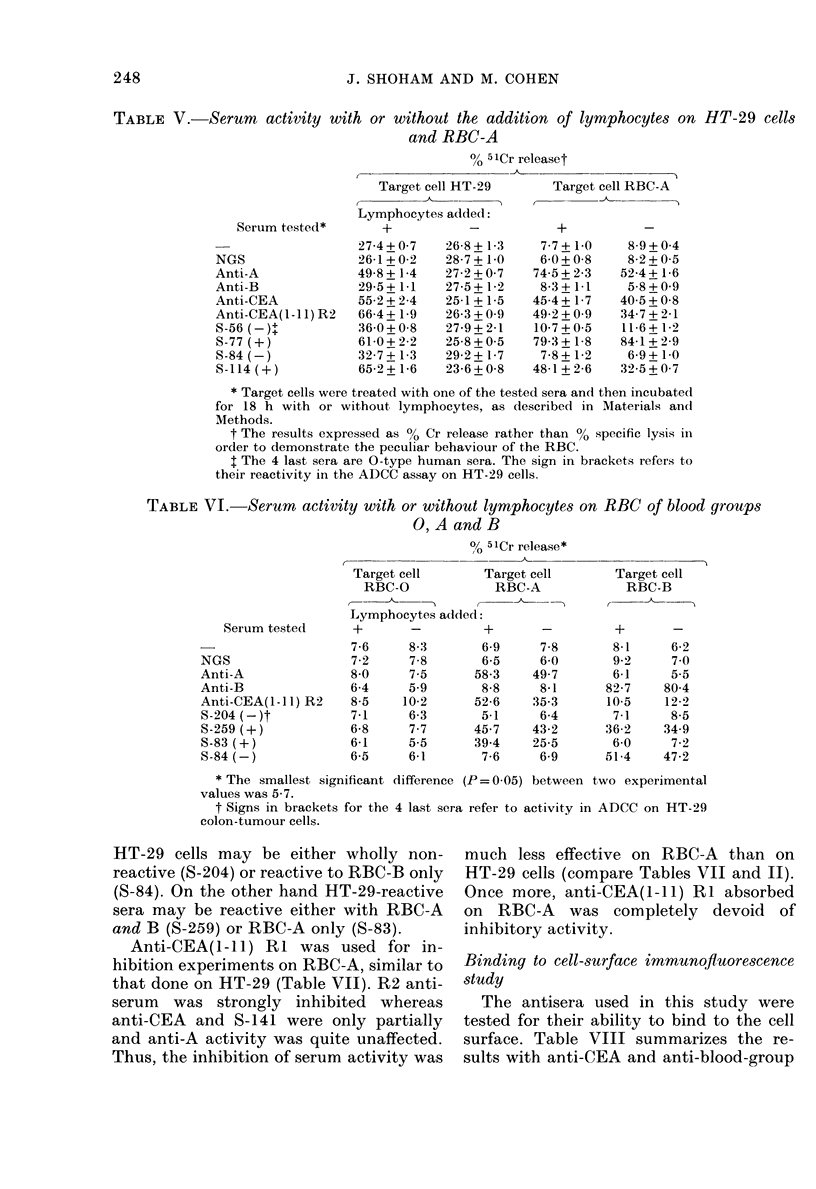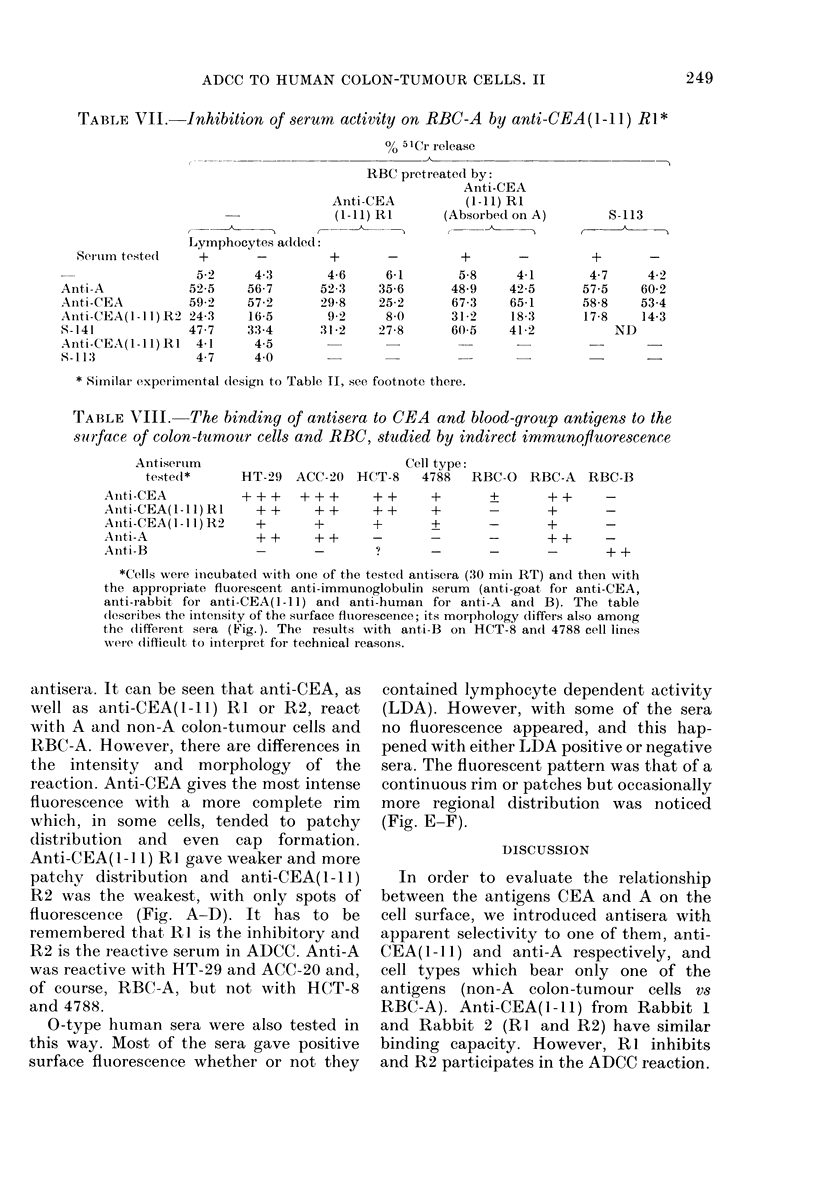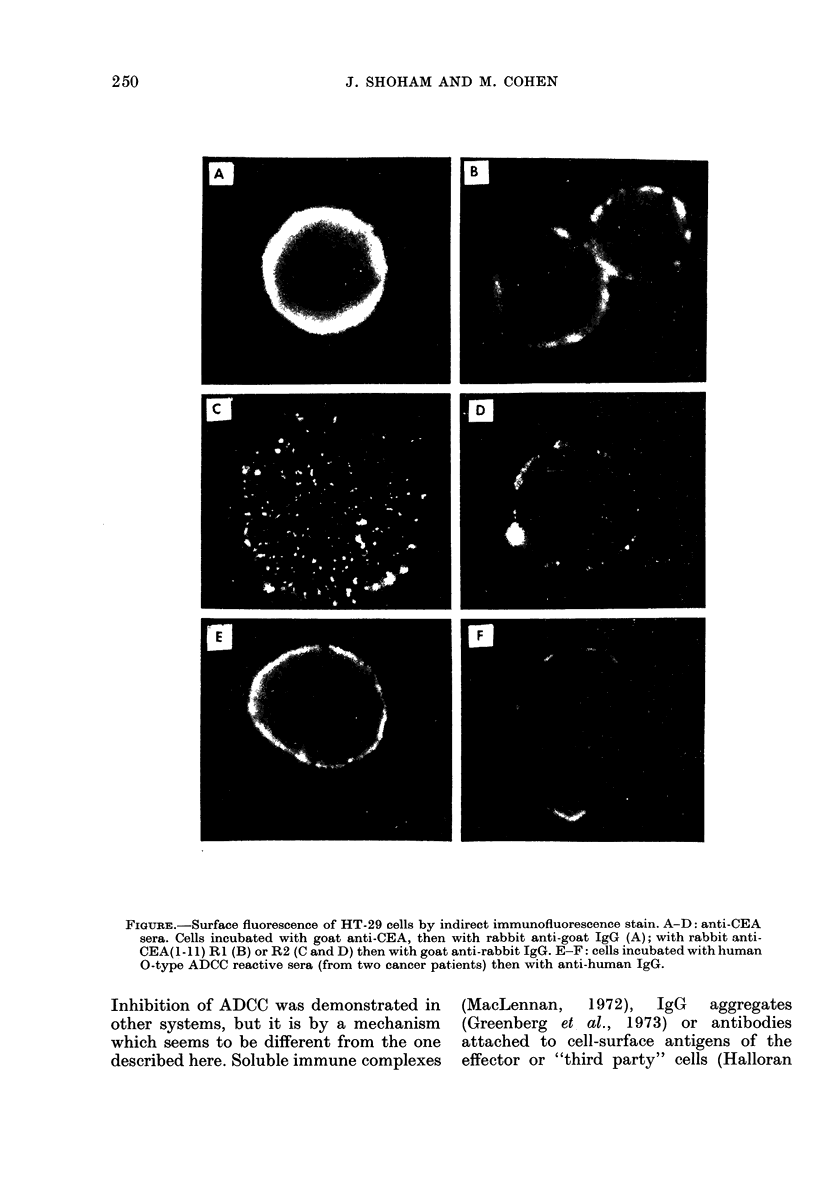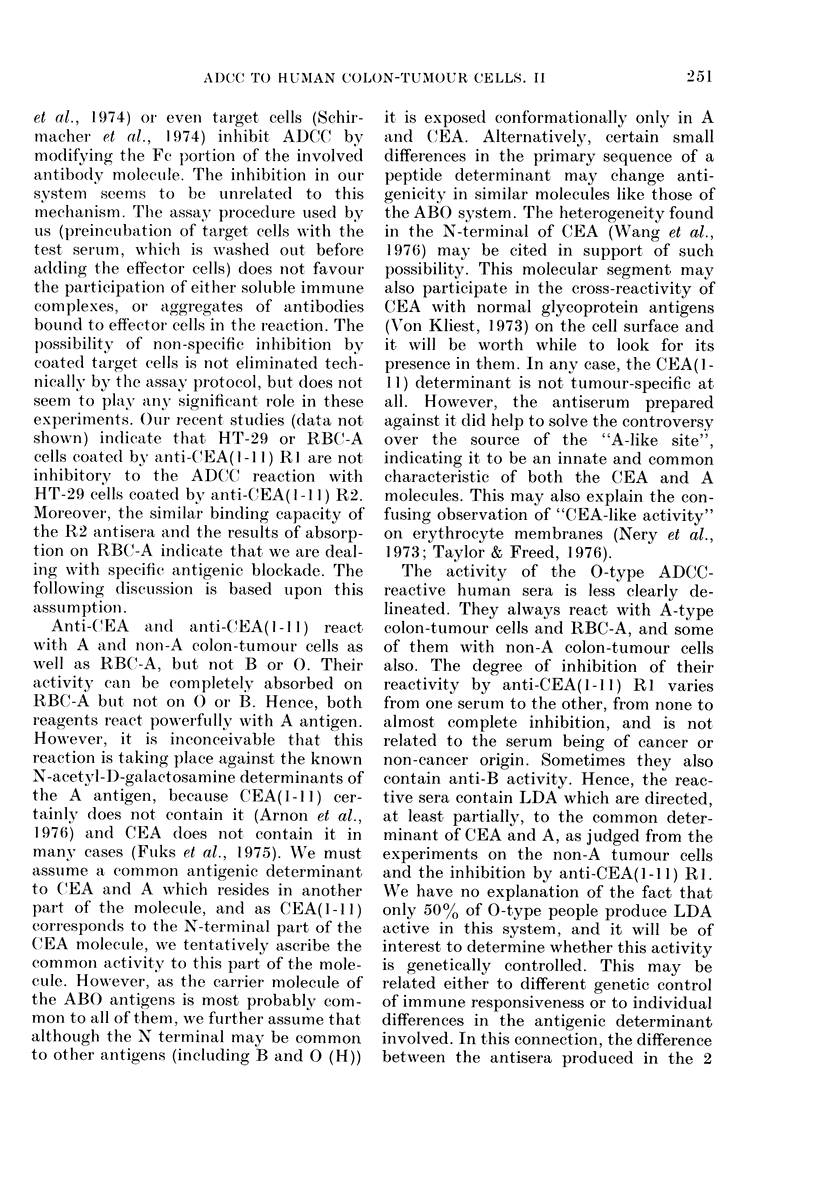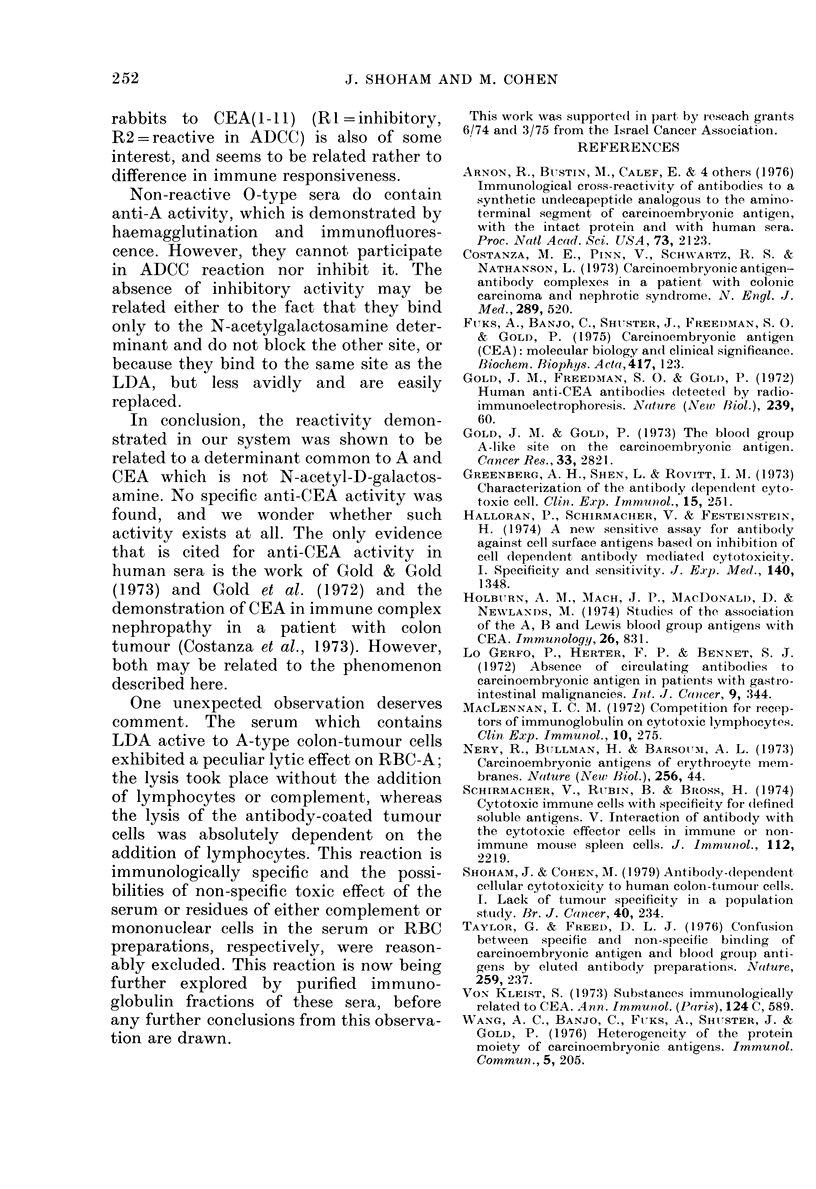# Antibody-dependent cellular cytotoxicity to human colon-tumour cells. II. Analysis of the antigens involved

**DOI:** 10.1038/bjc.1979.172

**Published:** 1979-08

**Authors:** J. Shoham, M. Cohen

## Abstract

**Images:**


*Br. J. Cancer* (1979) **40**, 244

### ANTIBODY-DEPENDENT CELLULAR CYTOTOXICITY TO HUMAN COLON-TUMOUR CELLS
### II. ANALYSIS OF THE ANTIGENS INVOLVED

J. SHOHAM AND M. COHEN

*From The Genetic Institute, Sheba Medical Center, Tel-Hashomer, Israel*

Received 30 November 1978 Accepted 12 April 1979

**Summary.—The relationship between carcinoembryonic antigen (CEA) and A antigenic determinants on the cell surface of colon-tumour cells was studied by the ADCC assay. Antiserum prepared in 2 rabbits to an undecapeptide analogous to the amino terminal of CEA(1-11) was found by us either to participate in (Rabbit 2) or specifically inhibit (Rabbit 1) ADCC. The binding spectra of these two antisera and of antiserum to the whole CEA molecule were similar. All of them react with A and non-A colon-tumour cells as well as red blood cells of Type A (RBC-A) and their activity was completely absorbed on RBC-A but not on B or O. O-type, ADCC-reactive human sera always react with A-type colon-tumour cells and RBC-A, and some of them with non-A colon-tumour cells also. The degree of inhibition of their reactivity by anti-CEA(1-11) R1 varied between sera, from none to almost a complete inhibition, and is not related to whether the serum is of cancer or non-cancer origin. Non-reactive O-type sera contain anti-A activity demonstrable by haemagglutination and immunofluorescence. However, they cannot participate in ADCC reaction nor inhibit it. The sera, which contain lymphocyte-dependent antibody to A-type colon-tumour cells, lysed RBC-A, without the addition of lymphocytes or complement, in an immunologically specific way. It is concluded that the reactivity seen in our ADCC system is related to a determinant common to A and CEA (and maybe to other normal cross-reacting antigens) which most probably resides in the amino terminal part of these molecules. This determinant elicits the production of lymphocyte-dependent antibodies in about 50% of people with blood group O. Thus, the amino terminal part of CEA is not a tumour-specific part of the CEA molecule. No specific anti-tumour activity was found in patients' serum by this method, and claims for its demonstration by other methods may well be related to the non-specific activity observed here.**

In the preceding article (Shoham & Cohen, 1979), the antibody-dependent cellular cytotoxicity (ADCC) reaction against human colon-tumour cells was used to test cellular and humoral activity in cancer patients and healthy controls. Definite humoral activity was found in 21% of the sera tested. However, this reactivity was not related to disease state but to blood-group O, and was apparently anti-A activity *per se*. On the other hand, "A-like activity" was demonstrated in purified preparations of CEA (Gold & Gold, 1973) and there is still controversy regarding its significance. Some people regard it as impurity due to separable A molecules (Lo Gerfo *et al.*, 1972), some relate it to the blood group of the patient and found respectively A, B or Le-specific determinants attached to the CEA molecule (Holburn *et al.*, 1974) and some claim that it is an innate characteristic of the CEA molecule, unrelated to the blood group of the patient (Gold & Gold,

Correspondence to: Dr Jacob Shoham, The Genetic Institute, Sheba Medical Center, Tel-Hashomer, Israel.

1973). The expression of this and other determinants when the CEA molecule is part of the cell surface is even less understood.

The present experiments were undertaken in order to study the relationship between A and CEA on the cell surface, and their relative contribution to the ADCC reaction. In addition, the possibility of demonstrating a more specific tumour-directed activity by using inhibitory antisera was also evaluated.

### MATERIALS AND METHODS

*Cell lines* were the same as in the previous study (Shoham & Cohen, 1979) but in addition, 2 other colon-tumour cell lines were used: HCT-8 and 4788, kindly given to us by Dr M Goldrosen from Roswell Park Memorial Institute, Buffalo, N.Y., U.S. Cell cultures were done as in the previous study.

*Sera.*—Goat anti-CEA serum was produced as in Shoham & Cohen (1979). Rabbit anti-CEA(1-11) was antiserum prepared by Dr Arnon and her group (Arnon *et al.*, 1976) against a synthetic peptide corresponding to the 11-amino-acid residues of the amino terminal portion in the sequence of CEA conjugated to BSA, and was kindly given to us by them. Anti-A and anti-B blood grouping serum, prepared from the blood of donors hyperimmunized with blood-group-specific substances, was purchased from Hyland (Div. Travenol Labs, CA). ADCC reactive and non-reactive human sera were selected from our previous study.

*Lymphocyte preparation and assay condition for ADCC* were as in the previous study.

*Serum absorption on RBC.*—Two volumes of serum with 1 volume of washed packed RBC were mixed and incubated at 37°C for 60 min. The procedure was repeated twice.

*Mixed haemadsorption* was used to determine the blood group of the cell lines. The tested cells were seeded in 24 cluster plates (Costar, Mass.) to get confluent monolayer cultures after overnight incubation. Then the cultures were washed and incubated with anti-A or anti-B serum for 30 min, washed again, and a 1% suspension of A or B RBC in PBS was added to the treated monolayers.

*Surface immunofluorescence.*—Cells were harvested and incubated with the tested serum for 30 min at room temperature then washed and incubated with fluorescein-conjugated antiserum to the tested serum.

### RESULTS

### *Determination of blood group of the colon-tumour cell lines*

This was done by 2 methods: mixed haemadsorption and immunofluorescence using commercial typing serum. It was found that ACC-20 and HT-29 had blood-group A antigen, and this was confirmed from the known blood group of the patients. Lines HCT-8 and 4788 were found to be non-A (O).

### *Absorption of anti-CEA on RBC*

Anti-CEA absorbed on RBC O or B retained about the same activity as non-absorbed serum. However, absorption on RBC-A eliminated ADCC activity almost completely. Similar results were obtained with ADCC-reactive O-type human serum (Table I). Thus, anti-CEA prepared against the whole CEA molecule is strongly cross-reacting with antigen A on RBC. There-

TABLE I.—*Specific absorption of ADCC activity of sera to A-type colon-tumour cell line by erythrocytes*

| | % Specific lysis (mean ± s.d.) | | | |
|---|---|---|---|---|
| | RBC absorption of serum | | | |
| Serum tested* | — | O | B | A |
| Anti-CEA | 50·4 ± 1·2 | 47·3 ± 1·8 | 45·0 ± 0·9 | 4·1 ± 0·3 |
| S-80 | 46·1 ± 0·7 | 40·5 ± 1·3 | 37·2 ± 2·1 | 7·4 ± 0·8 |

* Tested serum was used untreated or absorbed on O, B or A RBC, then tested for ADCC activity on HT-29 cells as described in Materials and Methods. S-80 is from a patient with adenocarcinoma of the pancreas, blood group O.

TABLE II.—*Sensitization for and inhibition of ADCC to A-type colon-tumour cell line by anti-CEA*(1-11) *sera*

| Serum tested | % Specific lysis (mean ± s.d.) Targets pretreated by — | Anti-CEA (1-11) R1* | Anti-CEA (1-11) R1 (Absorbed on A) | S-113 |
|---|---|---|---|---|
| Anti-A | 30·3 ± 1·8 | 14·2 ± 1·0 | 29·0 ± 2·7 | 32·5 ± 0·5 |
| Anti-CEA | 28·0 ± 2·1 | 4·1 ± 0·8 | 26·7 ± 1·2 | 30·8 ± 1·8 |
| Anti-CEA(1-11) R2* | 41·8 ± 0·7 | 5·3 ± 1·4 | 45·1 ± 0·4 | 42·2 ± 2·3 |
| S-141 | 26·5 ± 3·2 | 12·8 ± 2·1 | 24·2 ± 1·5 | 28·7 ± 0·7 |
| S-194 | 37·1 ± 0·3 | 11·7 ± 1·2 | ND | 33·8 ± 1·5 |
| Anti-CEA(1-11) R1 | 0 | — | — | — |
| S-113 | 0 | — | — | — |

* Anti-CEA(1-11) was prepared in 2 rabbits (R1 and R2). R1 antiserum exhibited inhibitory and R2 sensitization capability for ADCC. Target cells were incubated directly with tested serum or after incubation with R1 antiserum or S-113 (O type non-reactive human serum).

fore, for further experiments we introduced antisera prepared against a synthetic peptide corresponding to the 11 amino-acid residues of the amino terminal portion of the sequence of CEA:anti-CEA (1-11). This is obviously a different determinant from the known A determinant.

*ADCC with A-type colon-tumour cells*

Antisera prepared in 2 rabbits (R1 and R2) against CEA(1-11) conjugated to BSA, both found to be active in the bacteriophage-inactivation assay for CEA (Arnon *et al.*, 1976), were compared for the ADCC activity on A-type colon-tumour cells. Surprisingly, these 2 antisera differed completely in their ADCC activity. R1 antiserum was completely devoid of activity, whereas R2 antiserum was more potent than anti-CEA (Table II). However, when cells were incubated first with anti-CEA(1-11) R1 and then with anti-CEA(1-11) R2, the ADCC activity of the R2 antiserum was almost completely inhibited. Thus, Rabbit 1 produced antibodies that cannot participate in the ADCC reaction, but were avidly bound to CEA either in the phage assay or on the cell surface. Similar inhibitory activity of anti-CEA(1-11) R1 was also exerted on anti-CEA activity. On the other hand, the activity of anti-A, as well as that of reactive O-type human serum, was only partially inhibited. Moreover, if R1 antiserum was absorbed on RBC-A it completely lost its inhibitory activity to all the sera tested. A non-reactive O-type human serum (S-113) was completely without inhibitory activity, although it contains strongly agglutinating anti-A and anti-B (Table II).

We tested the possibility of using the inhibitory activity of anti-CEA(1-11) R1 for differentiating between cancer and non-cancer sera. The results (Table III) indicate that the degree of inhibition varies considerably from 0 to 90%. However, the degree of inhibition does not correlate with the disease state of the patient.

*ADCC with non-A colon-tumour cells*

Table IV compares the ADCC activity of several sera on A (ACC-20 and HT-29) and non-A (HCT-8 and 4788) colon-tumour cell lines. As expected, anti-A reacted only with the two A lines. Anti-CEA and anti-CEA(1-11) R2 reacted with all 4 lines, although the results with non-A cell lines are lower than those with A cells. It has to be emphasized, however, that non-immunological factors can contribute to such differences. The reactive O-type human sera differed in their ability to react with non-A cell lines; some react

TABLE III.—*Inhibition of ADCC activity of human sera by anti-CEA*(1-11) *R*1

| Serum tested* | Source | % Specific lysis — Targets pretreated by: — | % Specific lysis — Targets pretreated by: Anti-CEA (1-11) R1 | % Inhibition of lysis |
|---|---|---|---|---|
| S-167 | Non-cancer | 16·4 ± 1·0 | 17·1 ± 0·6 | 0 |
| S-114 | Breast ca. | 31·7 ± 2·8 | 27·0 ± 1·8 | 13 |
| S-135 | Melanoma | 20·5 ± 0·9 | 14·2 ± 1·1 | 30 |
| S-187 | Non-cancer | 26·3 ± 1·5 | 18·6 ± 0·5 | 32 |
| S-162 | Non-cancer | 21·0 ± 1·2 | 14·8 ± 0·8 | 33 |
| S-141 | Breast ca. | 26·6 ± 0·7 | 12·2 ± 1·0 | 53 |
| S-74 | Colon cancer | 46·5 ± 1·9 | 12·4 ± 0·6 | 73 |
| S-83 | Bladder ca. | 40·8 ± 1·1 | 5·3 ± 0·2 | 87 |
| S-184 | Non-cancer | 20·2 ± 1·2 | 2·0 ± 0·3 | 90 |

* ADCC-reactive blood-group O sera were tested for their activity on HT-29 cells. Results arranged according to the degree of inhibition exerted by the pretreatment.

TABLE IV.—*ADCC activity of different sera on A and non-A colon-tumour cells*

| Serum tested | % Specific lysis — Cell line tested: ACC-20 | HT-29 | HCT-8 | 4788 |
|---|---|---|---|---|
| Anti-A | 11·3 ± 0·4 | 15·5 ± 0·6 | 1·5 ± 0·8 | 0·7 ± 0·2 |
| Anti-CEA | 33·4 ± 0·7 | 24·0 ± 1·3 | 17·8 ± 1·1 | 14·6 ± 0·5 |
| Anti-CEA(1-11) R2 | 42·1 ± 1·8 | 21·1 ± 1·5 | 10·8 ± 0·6 | 8·5 ± 0·8 |
| S-52 | 18·7 ± 1·1 | 21·3 ± 0·9 | 16·3 ± 0·7 | 4·1 ± 0·4 |
| S-61 | 2·1 ± 0·5 | 4·3 ± 0·8 | 1·5 ± 1·0 | 1·8 ± 0·7 |
| S-74 | 37·2 ± 1·5 | 26·7 ± 2·3 | 3·3 ± 0·4 | ND |
| S-187 | 18·8 ± 2·1 | 23·2 ± 0·8 | 15·4 ± 2·1 | 21·8 ± 1·3 |
| S-176 | 35·9 ± 1·0 | 16·2 ± 1·2 | 4·5 ± 0·7 | 2·8 ± 1·0 |

with only one of these lines (*i.e.* S-52), some with the 2 lines (S-197) and some do not react at all (S-74, S-176).

*ADCC with RBC*

In the first experiments we compared the ADCC activity of the different sera on HT-29 and RBC-A (Table V). The spontaneous release (with or without preincubation in normal goat serum, NGS) was much lower with RBC than with HT-29. The presence of lymphocytes did not change the degree of $^{51}Cr$ release. However, in the presence of reactive sera, a remarkable difference was noticed between the HT-29 cells and RBC. The presence of the heat-inactivated serum alone was enough to cause $^{51}Cr$ release from RBC to a degree equal or slightly lower than with lymphocytes, whereas with HT-29 the activity of the serum was expressed only in the presence of lymphocytes. Both anti-CEA and anti-CEA(1-11) R2 were reactive with RBC-A to about the same degree. Only human sera which reacted in ADCC against tumour cells were reactive against RBC-A without need for lymphocyte assistance, or participation of complement (inactivated serum; some of the experiments in the presence of carrageenan).

In the following tables the results will be expressed as % $^{51}Cr$ release rather than % specific lysis, in order to account for the antibody effect *per se*.

The immunological specificity of the reactions was tested by using O, A and B-type RBC (Table VI). As expected, commercial anti-A and anti-B preparations reacted only with A or B cells, respectively, with essentially the same results with or without adding lymphocytes. Anti-CEA(1-11) R2 and anti-CEA reacted with RBC-A only. The most interesting results were with O-type human sera. Sera which do not react with

TABLE V.—*Serum activity with or without the addition of lymphocytes on HT-29 cells and RBC-A*

| Serum tested* | % $^{51}$Cr release† Target cell HT-29 Lymphocytes added: + | − | Target cell RBC-A + | − |
|---|---|---|---|---|
| — | 27·4 ± 0·7 | 26·8 ± 1·3 | 7·7 ± 1·0 | 8·9 ± 0·4 |
| NGS | 26·1 ± 0·2 | 28·7 ± 1·0 | 6·0 ± 0·8 | 8·2 ± 0·5 |
| Anti-A | 49·8 ± 1·4 | 27·2 ± 0·7 | 74·5 ± 2·3 | 52·4 ± 1·6 |
| Anti-B | 29·5 ± 1·1 | 27·5 ± 1·2 | 8·3 ± 1·1 | 5·8 ± 0·9 |
| Anti-CEA | 55·2 ± 2·4 | 25·1 ± 1·5 | 45·4 ± 1·7 | 40·5 ± 0·8 |
| Anti-CEA(1-11) R2 | 66·4 ± 1·9 | 26·3 ± 0·9 | 49·2 ± 0·9 | 34·7 ± 2·1 |
| S-56 (−)‡ | 36·0 ± 0·8 | 27·9 ± 2·1 | 10·7 ± 0·5 | 11·6 ± 1·2 |
| S-77 (+) | 61·0 ± 2·2 | 25·8 ± 0·5 | 79·3 ± 1·8 | 84·1 ± 2·9 |
| S-84 (−) | 32·7 ± 1·3 | 29·2 ± 1·7 | 7·8 ± 1·2 | 6·9 ± 1·0 |
| S-114 (+) | 65·2 ± 1·6 | 23·6 ± 0·8 | 48·1 ± 2·6 | 32·5 ± 0·7 |

* Target cells were treated with one of the tested sera and then incubated for 18 h with or without lymphocytes, as described in Materials and Methods.

† The results expressed as % Cr release rather than % specific lysis in order to demonstrate the peculiar behaviour of the RBC.

‡ The 4 last sera are O-type human sera. The sign in brackets refers to their reactivity in the ADCC assay on HT-29 cells.

TABLE VI.—*Serum activity with or without lymphocytes on RBC of blood groups O, A and B*

| Serum tested | % $^{51}$Cr release* Target cell RBC-O Lymphocytes added: + | − | Target cell RBC-A + | − | Target cell RBC-B + | − |
|---|---|---|---|---|---|---|
| — | 7·6 | 8·3 | 6·9 | 7·8 | 8·1 | 6·2 |
| NGS | 7·2 | 7·8 | 6·5 | 6·0 | 9·2 | 7·0 |
| Anti-A | 8·0 | 7·5 | 58·3 | 49·7 | 6·1 | 5·5 |
| Anti-B | 6·4 | 5·9 | 8·8 | 8·1 | 82·7 | 80·4 |
| Anti-CEA(1-11) R2 | 8·5 | 10·2 | 52·6 | 35·3 | 10·5 | 12·2 |
| S-204 (−)† | 7·1 | 6·3 | 5·1 | 6·4 | 7·1 | 8·5 |
| S-259 (+) | 6·8 | 7·7 | 45·7 | 43·2 | 36·2 | 34·9 |
| S-83 (+) | 6·1 | 5·5 | 39·4 | 25·5 | 6·0 | 7·2 |
| S-84 (−) | 6·5 | 6·1 | 7·6 | 6·9 | 51·4 | 47·2 |

* The smallest significant difference ($P = 0·05$) between two experimental values was 5·7.

† Signs in brackets for the 4 last sera refer to activity in ADCC on HT-29 colon-tumour cells.

HT-29 cells may be either wholly non-reactive (S-204) or reactive to RBC-B only (S-84). On the other hand HT-29-reactive sera may be reactive either with RBC-A *and* B (S-259) or RBC-A only (S-83).

Anti-CEA(1-11) R1 was used for inhibition experiments on RBC-A, similar to that done on HT-29 (Table VII). R2 antiserum was strongly inhibited whereas anti-CEA and S-141 were only partially and anti-A activity was quite unaffected. Thus, the inhibition of serum activity was much less effective on RBC-A than on HT-29 cells (compare Tables VII and II). Once more, anti-CEA(1-11) R1 absorbed on RBC-A was completely devoid of inhibitory activity.

*Binding to cell-surface immunofluorescence study*

The antisera used in this study were tested for their ability to bind to the cell surface. Table VIII summarizes the results with anti-CEA and anti-blood-group

TABLE VII.—*Inhibition of serum activity on RBC-A by anti-CEA*(1-11) *R*1*

| | % $^{51}$Cr release | | | | | | | |
|---|---|---|---|---|---|---|---|---|
| | RBC pretreated by: | | | | | | | |
| | — | | Anti-CEA (1-11) R1 | | Anti-CEA (1-11) R1 (Absorbed on A) | | S-113 | |
| | Lymphocytes added: | | | | | | | |
| Serum tested | + | − | + | − | + | − | + | − |
| — | 5·2 | 4·3 | 4·6 | 6·1 | 5·8 | 4·1 | 4·7 | 4·2 |
| Anti-A | 52·5 | 56·7 | 52·3 | 35·6 | 48·9 | 42·5 | 57·5 | 60·2 |
| Anti-CEA | 59·2 | 57·2 | 29·8 | 25·2 | 67·3 | 65·1 | 58·8 | 53·4 |
| Anti-CEA(1-11) R2 | 24·3 | 16·5 | 9·2 | 8·0 | 31·2 | 18·3 | 17·8 | 14·3 |
| S-141 | 47·7 | 33·4 | 31·2 | 27·8 | 60·5 | 41·2 | ND | |
| Anti-CEA(1-11) R1 | 4·1 | 4·5 | — | — | — | — | — | — |
| S-113 | 4·7 | 4·0 | — | — | — | — | — | — |

* Similar experimental design to Table II, see footnote there.

TABLE VIII.—*The binding of antisera to CEA and blood-group antigens to the surface of colon-tumour cells and RBC, studied by indirect immunofluorescence*

| Antiserum tested* | Cell type: HT-29 | ACC-20 | HCT-8 | 4788 | RBC-O | RBC-A | RBC-B |
|---|---|---|---|---|---|---|---|
| Anti-CEA | + + + | + + + | + + | + | ± | + + | − |
| Anti-CEA(1-11) R1 | + + | + + | + + | + | − | + | − |
| Anti-CEA(1-11) R2 | + | + | + | ± | − | + | − |
| Anti-A | + + | + + | − | − | − | + + | − |
| Anti-B | − | − | ? | − | − | − | + + |

*Cells were incubated with one of the tested antisera (30 min RT) and then with the appropriate fluorescent anti-immunoglobulin serum (anti-goat for anti-CEA, anti-rabbit for anti-CEA(1-11) and anti-human for anti-A and B). The table describes the intensity of the surface fluorescence; its morphology differs also among the different sera (Fig.). The results with anti-B on HCT-8 and 4788 cell lines were difficult to interpret for technical reasons.

antisera. It can be seen that anti-CEA, as well as anti-CEA(1-11) R1 or R2, react with A and non-A colon-tumour cells and RBC-A. However, there are differences in the intensity and morphology of the reaction. Anti-CEA gives the most intense fluorescence with a more complete rim which, in some cells, tended to patchy distribution and even cap formation. Anti-CEA(1-11) R1 gave weaker and more patchy distribution and anti-CEA(1-11) R2 was the weakest, with only spots of fluorescence (Fig. A–D). It has to be remembered that R1 is the inhibitory and R2 is the reactive serum in ADCC. Anti-A was reactive with HT-29 and ACC-20 and, of course, RBC-A, but not with HCT-8 and 4788.

O-type human sera were also tested in this way. Most of the sera gave positive surface fluorescence whether or not they contained lymphocyte dependent activity (LDA). However, with some of the sera no fluorescence appeared, and this happened with either LDA positive or negative sera. The fluorescent pattern was that of a continuous rim or patches but occasionally more regional distribution was noticed (Fig. E–F).

### DISCUSSION

In order to evaluate the relationship between the antigens CEA and A on the cell surface, we introduced antisera with apparent selectivity to one of them, anti-CEA(1-11) and anti-A respectively, and cell types which bear only one of the antigens (non-A colon-tumour cells *vs* RBC-A). Anti-CEA(1-11) from Rabbit 1 and Rabbit 2 (R1 and R2) have similar binding capacity. However, R1 inhibits and R2 participates in the ADCC reaction.

FIGURE.—Surface fluorescence of HT-29 cells by indirect immunofluorescence stain. A–D: anti-CEA sera. Cells incubated with goat anti-CEA, then with rabbit anti-goat IgG (A); with rabbit anti-CEA(1-11) R1 (B) or R2 (C and D) then with goat anti-rabbit IgG. E–F: cells incubated with human O-type ADCC reactive sera (from two cancer patients) then with anti-human IgG.

Inhibition of ADCC was demonstrated in other systems, but it is by a mechanism which seems to be different from the one described here. Soluble immune complexes (MacLennan, 1972), IgG aggregates (Greenberg *et al.*, 1973) or antibodies attached to cell-surface antigens of the effector or "third party" cells (Halloran

*et al.*, 1974) or even target cells (Schirmacher *et al.*, 1974) inhibit ADCC by modifying the Fc portion of the involved antibody molecule. The inhibition in our system seems to be unrelated to this mechanism. The assay procedure used by us (preincubation of target cells with the test serum, which is washed out before adding the effector cells) does not favour the participation of either soluble immune complexes, or aggregates of antibodies bound to effector cells in the reaction. The possibility of non-specific inhibition by coated target cells is not eliminated technically by the assay protocol, but does not seem to play any significant role in these experiments. Our recent studies (data not shown) indicate that HT-29 or RBC-A cells coated by anti-CEA(1-11) R1 are not inhibitory to the ADCC reaction with HT-29 cells coated by anti-CEA(1-11) R2. Moreover, the similar binding capacity of the R2 antisera and the results of absorption on RBC-A indicate that we are dealing with specific antigenic blockade. The following discussion is based upon this assumption.

Anti-CEA and anti-CEA(1-11) react with A and non-A colon-tumour cells as well as RBC-A, but not B or O. Their activity can be completely absorbed on RBC-A but not on O or B. Hence, both reagents react powerfully with A antigen. However, it is inconceivable that this reaction is taking place against the known N-acetyl-D-galactosamine determinants of the A antigen, because CEA(1-11) certainly does not contain it (Arnon *et al.*, 1976) and CEA does not contain it in many cases (Fuks *et al.*, 1975). We must assume a common antigenic determinant to CEA and A which resides in another part of the molecule, and as CEA(1-11) corresponds to the N-terminal part of the CEA molecule, we tentatively ascribe the common activity to this part of the molecule. However, as the carrier molecule of the ABO antigens is most probably common to all of them, we further assume that although the N terminal may be common to other antigens (including B and O (H)) it is exposed conformationally only in A and CEA. Alternatively, certain small differences in the primary sequence of a peptide determinant may change antigenicity in similar molecules like those of the ABO system. The heterogeneity found in the N-terminal of CEA (Wang *et al.*, 1976) may be cited in support of such possibility. This molecular segment may also participate in the cross-reactivity of CEA with normal glycoprotein antigens (Von Kliest, 1973) on the cell surface and it will be worth while to look for its presence in them. In any case, the CEA(1-11) determinant is not tumour-specific at all. However, the antiserum prepared against it did help to solve the controversy over the source of the "A-like site", indicating it to be an innate and common characteristic of both the CEA and A molecules. This may also explain the confusing observation of "CEA-like activity" on erythrocyte membranes (Nery *et al.*, 1973; Taylor & Freed, 1976).

The activity of the O-type ADCC-reactive human sera is less clearly delineated. They always react with A-type colon-tumour cells and RBC-A, and some of them with non-A colon-tumour cells also. The degree of inhibition of their reactivity by anti-CEA(1-11) R1 varies from one serum to the other, from none to almost complete inhibition, and is not related to the serum being of cancer or non-cancer origin. Sometimes they also contain anti-B activity. Hence, the reactive sera contain LDA which are directed, at least partially, to the common determinant of CEA and A, as judged from the experiments on the non-A tumour cells and the inhibition by anti-CEA(1-11) R1. We have no explanation of the fact that only 50% of O-type people produce LDA active in this system, and it will be of interest to determine whether this activity is genetically controlled. This may be related either to different genetic control of immune responsiveness or to individual differences in the antigenic determinant involved. In this connection, the difference between the antisera produced in the 2

rabbits to CEA(1-11) (R1 = inhibitory, R2 = reactive in ADCC) is also of some interest, and seems to be related rather to difference in immune responsiveness.

Non-reactive O-type sera do contain anti-A activity, which is demonstrated by haemagglutination and immunofluorescence. However, they cannot participate in ADCC reaction nor inhibit it. The absence of inhibitory activity may be related either to the fact that they bind only to the N-acetylgalactosamine determinant and do not block the other site, or because they bind to the same site as the LDA, but less avidly and are easily replaced.

In conclusion, the reactivity demonstrated in our system was shown to be related to a determinant common to A and CEA which is not N-acetyl-D-galactosamine. No specific anti-CEA activity was found, and we wonder whether such activity exists at all. The only evidence that is cited for anti-CEA activity in human sera is the work of Gold & Gold (1973) and Gold *et al.* (1972) and the demonstration of CEA in immune complex nephropathy in a patient with colon tumour (Costanza *et al.*, 1973). However, both may be related to the phenomenon described here.

One unexpected observation deserves comment. The serum which contains LDA active to A-type colon-tumour cells exhibited a peculiar lytic effect on RBC-A; the lysis took place without the addition of lymphocytes or complement, whereas the lysis of the antibody-coated tumour cells was absolutely dependent on the addition of lymphocytes. This reaction is immunologically specific and the possibilities of non-specific toxic effect of the serum or residues of either complement or mononuclear cells in the serum or RBC preparations, respectively, were reasonably excluded. This reaction is now being further explored by purified immunoglobulin fractions of these sera, before any further conclusions from this observation are drawn.

This work was supported in part by reseach grants 6/74 and 3/75 from the Israel Cancer Association.

REFERENCES

Arnon, R., Bustin, M., Calef, E. & 4 others (1976) Immunological cross-reactivity of antibodies to a synthetic undecapeptide analogous to the amino-terminal segment of carcinoembryonic antigen, with the intact protein and with human sera. *Proc. Natl Acad. Sci. USA*, **73,** 2123.

Costanza, M. E., Pinn, V., Schwartz, R. S. & Nathanson, L. (1973) Carcinoembryonic antigen–antibody complexes in a patient with colonic carcinoma and nephrotic syndrome. *N. Engl. J. Med.*, **289,** 520.

Fuks, A., Banjo, C., Shuster, J., Freedman, S. O. & Gold, P. (1975) Carcinoembryonic antigen (CEA): molecular biology and clinical significance. *Biochem. Biophys. Acta*, **417,** 123.

Gold, J. M., Freedman, S. O. & Gold, P. (1972) Human anti-CEA antibodies detected by radioimmunoelectrophoresis. *Nature* (*New Biol.*), **239,** 60.

Gold, J. M. & Gold, P. (1973) The blood group A-like site on the carcinoembryonic antigen. *Cancer Res.*, **33,** 2821.

Greenberg, A. H., Shen, L. & Rovitt, I. M. (1973) Characterization of the antibody dependent cytotoxic cell. *Clin. Exp. Immunol.*, **15,** 251.

Halloran, P., Schirmacher, V. & Festeinstein, H. (1974) A new sensitive assay for antibody against cell surface antigens based on inhibition of cell dependent antibody mediated cytotoxicity. I. Specificity and sensitivity. *J. Exp. Med.*, **140,** 1348.

Holburn, A. M., Mach, J. P., MacDonald, D. & Newlands, M. (1974) Studies of the association of the A, B and Lewis blood group antigens with CEA. *Immunology*, **26,** 831.

Lo Gerfo, P., Herter, F. P. & Bennet, S. J. (1972) Absence of circulating antibodies to carcinoembryonic antigen in patients with gastrointestinal malignancies. *Int. J. Cancer*, **9,** 344.

MacLennan, I. C. M. (1972) Competition for receptors of immunoglobulin on cytotoxic lymphocytes. *Clin Exp. Immunol.*, **10,** 275.

Nery, R., Bullman, H. & Barsoum, A. L. (1973) Carcinoembryonic antigens of erythrocyte membranes. *Nature* (*New Biol.*), **256,** 44.

Schirmacher, V., Rubin, B. & Bross, H. (1974) Cytotoxic immune cells with specificity for defined soluble antigens. V. Interaction of antibody with the cytotoxic effector cells in immune or non-immune mouse spleen cells. *J. Immunol.*, **112,** 2219.

Shoham, J. & Cohen, M. (1979) Antibody-dependent cellular cytotoxicity to human colon-tumour cells. I. Lack of tumour specificity in a population study. *Br. J. Cancer*, **40,** 234.

Taylor, G. & Freed, D. L. J. (1976) Confusion between specific and non-specific binding of carcinoembryonic antigen and blood group antigens by eluted antibody preparations. *Nature*, **259,** 237.

Von Kleist, S. (1973) Substances immunologically related to CEA. *Ann. Immunol.* (*Paris*), **124** C, 589.

Wang, A. C., Banjo, C., Fuks, A., Shuster, J. & Gold, P. (1976) Heterogeneity of the protein moiety of carcinoembryonic antigens. *Immunol. Commun.*, **5,** 205.